# Machine learning classifiers for predicting 3-year progression-free survival and overall survival in patients with gliomas after surgery

**DOI:** 10.7150/jca.52183

**Published:** 2021-01-15

**Authors:** Bin Zhang, Jing Yan, Weiqi Chen, Yuhao Dong, Lu Zhang, Xiaokai Mo, Qiuying Chen, Jingliang Cheng, Xianzhi Liu, Weiwei Wang, Zhenyu Zhang, Shuixing Zhang

**Affiliations:** 1 Department of Radiology, The First Affiliated Hospital of Jinan University, Guangzhou, Guangdong, China.; 2 Department of MRI, The First Affiliated Hospital of Zhengzhou University, Zhengzhou, China.; 3 Big Data Decision Institute, Jinan University, Guangzhou, Guangdong, China.; 4 School of management, Jinan University. Department of Catheterization Lab, Guangdong Cardiovascular Institute, Guangdong, Provincial Key Laboratory of South China; 5 Structural Heart Disease, Guangdong Provincial; People's Hospital/Guangdong Academy of Medical Sciences, Guangzhou, Guangdong, P.R. China.; 6 Department of Neurosurgery, The First Affiliated Hospital of Zhengzhou University, Zhengzhou, China.; 7 Department of Pathology, The First Affiliated Hospital of Zhengzhou University, Zhengzhou, China.

**Keywords:** gliomas, molecular biomarkers, machine learning, progression-free survival, overall survival

## Abstract

**Background:** To develop machine-learning based models to predict the progression-free survival (PFS) and overall survival (OS) in patients with gliomas and explore the effect of different feature selection methods on the prediction.

**Methods:** We included 505 patients (training cohort, n = 354; validation cohort, n = 151) with gliomas between January 1, 2011 and December 31, 2016. The clinical, neuroimaging, and molecular genetic data of patients were retrospectively collected. The multi-causes discovering with structure learning (McDSL) algorithm, least absolute shrinkage and selection operator regression (LASSO), and Cox proportional hazards regression model were employed to discover the predictors for 3-year PFS and OS, respectively. Eight machine learning classifiers with 5-fold cross-validation were developed to predict 3-year PFS and OS. The area under the curve (AUC) was used to evaluate the prognostic performance of classifiers.

**Results:** McDSL identified four causal factors (tumor location, WHO grade, histologic type, and molecular genetic group) for 3-year PFS and OS, whereas LASSO and Cox identified wide-range number of factors associated with 3-year PFS and OS. The performance of each machine learning classifier based on McDSL, LASSO, and Cox was not significantly different. Logistic regression yielded the optimal performance in predicting 3-year PFS based on the McDSL (AUC, 0.872, 95% confidence interval [CI]: 0.828-0.916) and 3-year OS based on the LASSO (AUC, 0.901, 95% CI: 0.861-0.940).

**Conclusions:** McDSL is more reproducible than LASSO and Cox model in the feature selection process. Logistic regression model may have the highest performance in predicting 3-year PFS and OS of gliomas.

## Introduction

Gliomas are the most common primary malignant central nervous system in adults 1, which account for 80% of malignant brain tumors 2. Gliomas are associated with substantial mortality and morbidity 3. The prediction of clinical behavior, response to treatment, and survival outcome of glioma is challenging. Prognostic assessment of gliomas is very crucial for patient counseling, treatment strategy planning, and disease monitoring 4.

Low-grade gliomas typically affect younger adults and carry a favorable prognosis, while high-grade tumors generally have the worst progression-free survival (PFS) and overall survival (OS). The OS for WHO grades II, III, and IV astrocytomas is approximately 6-8 years, 2 years, and 15 months, respectively 5. Additional factors associated with survival outcome are age, sex, Karnofsky Performance Status (KPS), tumor morphology, extent of tumor resection, and treatment methods (surgery, radiotherapy, and chemotherapy) 5-7. However, the prognostic value of these factors depends on the tumor grade and histological subtype. What's more, some of these factors are correlated with or confounded by other factors, and higher tumor grade is correlated with more advanced age. The influence of extent of resection on survival could be confounded by tumor location, whether it is resectable or non-resectable, and/or by clinical judgement 8.

The tumor biology is associated with the status of different molecular markers, such as isocitrate dehydrogenase (IDH), 1p/19q codeletion, and telomerase reverse transcriptase (TERT) mutation 9. As compared with the WHO 2007 classification, the new classification announced by the WHO in 2016 recognized several new entities of diffuse glioma based on genotypes (IDH mutation and 1p/19q codeletion) in addition to the histologic phenotypes of tumors 10-12. On the basis of previous studies of tumor biology, Eckel-Passow JE et al. proposed five molecular groups according to the three biomarkers: triple-positive (mutations in both TERT and IDH plus 1p/19q codeletion), mutations in both TERT and IDH, mutation in IDH only, mutation in TERT only, and triple-negative 9. Recent studies on gliomas using The Cancer Genome Atlas database have revealed the association of IDH mutation, 1p/19q codeletion, and TERT promoter mutation with OS 9, 13, 14. Favorable prognosis has been observed in tumors with IDH mutation and/or 1p/19q codeletion but poor prognosis in those with TERT promoter mutation.

The success of precision oncology relies on accurately categorizing patients on the basis of their prognostic characteristics. Therefore, we aimed to develop machine learning based clinical models to predict the 3-year PFS and OS among patients with gliomas and explore the effect of different feature selection methods on the predictive performance of machine learning classifiers.

## Materials and Methods

### Patient population and data collection

**Figure 1:**
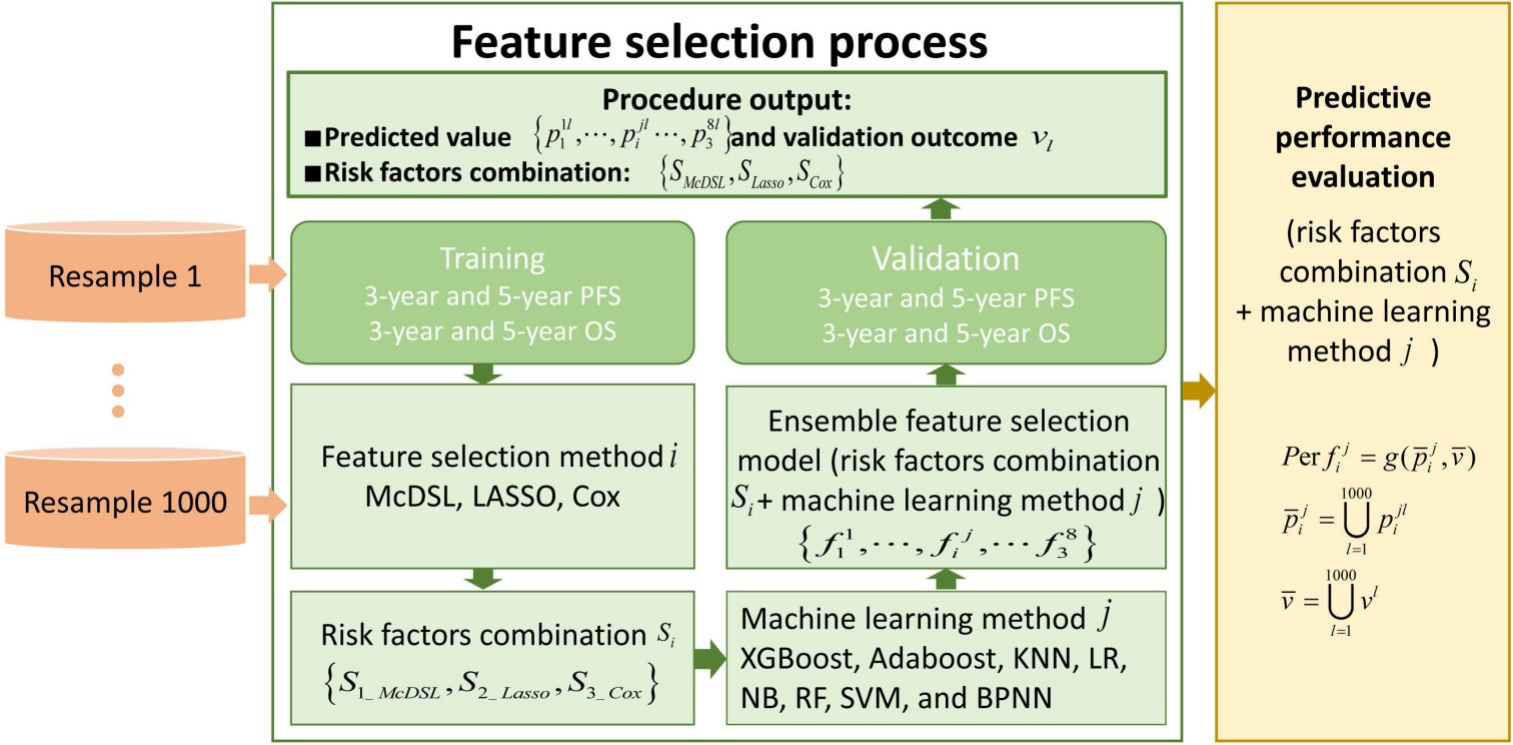
The workflow of this study. To evaluate the performance of each experiment of feature selection method *i* and machine learning method *j*, predicted value and validation outcome of 1000 times resample were combined into 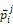 and 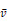 . 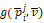 denotes that evaluate equation of predictive performance.

This retrospective study was approved by the local Institutional Review Board before data collection and analysis, which waived the requirement for written consent. We included a total of 505 consecutive patients at the neurosurgery department between January 1, 2011 and December 31, 2016. Inclusion criteria were as follows: (i) patients aged ≥18 at the time of first surgery; (ii) patients were treated by surgical resection with or without postoperative chemoradiotherapy; (iii) patients who had infiltrative glioma of histologic grade II, III, or IV. Grade I tumor (pilocytic astrocytoma) are clinically and pathologically distant and therefore was not included; (iv) patients were followed up ≥36 months; and v) patients had information of molecular alterations, including IDH mutation and 1p/19q codeletion, and TERT promoter mutation. Those patients with recurrent gliomas or underwent biopsy only were excluded. The following data were obtained for each patient: age at initial diagnosis, sex, KPS on admission, tumor location, histologic type, WHO grade, extent of surgical resection of tumor, radiotherapy, chemotherapy, molecular alterations, and survival outcomes. All of the cases were evaluated by a neuro-radiologist and a neuro-oncologist. Disagreements between the two observers were resolved by consensus and, if necessary, discussion with a third observer. The extent of resection was determined by analysis of pre- and post-operatively acquired cranial MRI scans 7. Ideally, the post-surgical MRI was performed up to 72 hours after surgery. Identification of IDH1 mutation was performed by pyrosequencing of an 88-bp-long fragment of the IDH1 gene with the mutation hotspot at codon 132 15, 16. For IDH2 mutations, pyrosequencing was performed on an 83-bp-long fragment of the IDH2 gene with the mutation hotspot at codon 172 15, 16. IDH1 and IDH2 are very similar to each other and hereafter collectively referred as IDH. 1p/19q codeletion was analyzed by fluorescence in situ hybridisation according to standard protocols 17. Detection of TERT promoter was performed by direct sequencing as previously reported 18. Based on the status of IDH mutation, 1p/19q codeletion, and TERT promoter mutation, gliomas can be classified into five molecular groups 9: triple-positive (mutations in both TERT and IDH plus 1p/19q codeletion), mutations in both TERT and IDH, mutation in IDH only, triple-negative, and mutation in TERT only. The primary outcomes were 3-year PFS and OS. PFS was defined as the interval between date of initial diagnosis (date of first surgery) and either disease progression or death, censored at the last follow-up visit. OS was defined as the interval from the date of surgery until date of death, censored at the last follow-up visit. Figure 1 illustrates the workflow of this study.

### Feature selection methods

Potential predictors including age at initial diagnosis, sex, KPS on admission, tumor location, histologic type, WHO grade, extent of surgical resection of tumor, radiotherapy, chemotherapy, IDH1 mutation, IDH2 mutation, IDH mutation, 1p deletion, 19q deletion, 1p19q codeletion, TERT promoter mutation, and molecular groups. The multi-causes discovering with structure learning (McDSL) algorithm, the least absolute shrinkage and selection operator regression (LASSO), and Cox proportional hazards regression model were applied to select the factors.

### Multi-causes discovering with structure learning algorithm

In this study, the multi-causes discovering with McDSL algorithm 19, 20 was used as a causal assumption inferring method to discover the risk factors combination of PFS and OS of gliomas patient.

The dataset has 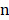 factors *X* = {*x*_1_*, …, x_n_*} and one outcome 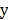 (label). The McDSL discovers the risk factors combination through two phases: 1) search and transform each factors combination *S* = {*x_i_,…, x_j_*} into a factor *x_s_,* 2) infer the causal relationship between *x_s_* and label *y* with additive noise model (ANM).

Firstly, calculating the correlation R of each factor *x_i_* and label 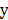 with their joint distribution, as shown in the following equation.


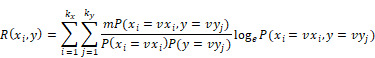


Among which, *R(x_i_,y)* is the correlation of *x_i_* and *y*, *m* is the sample size of data, *vx_i_* is the *i*-th value of *x_i_*, *k_x_* is the number of values of *x_i_*, *vy_j_* is the *j*-th value of *y*,* k_y_* is the number of values of *y*, *P(x_i_ = vx_i_, y = vy_j_)* is the joint distribution.

Secondly, selecting the combination *S* with described correlation R, and transforming it into factor *x_s_* with the value combination of factors. The scale *|S|* was increased from 1 to *n.*

Finally, ANM was employed to infer the causal relationship between each *x_s_* and label *y.* ANM was presented to deal with the causal relationship of binary factors, and it performed well in handling binary discrete synthetic data (accuracy >93%) 21 and inferring the relationship between transformed factor and label (accuracy >90% in the most of combination scales) 20. In the process of causal relationship assumption inferring of ANM, an additive noise was considered as existed, which could been undiscovered or unrecorded factor, missing data, and human error, etc. The formulae are shown as follows:


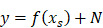
, and 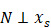


Of which, *f* is the mapping from *x_s_* to *y*, *N* is noise, *N*
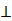
*x_s_* means *N* and *x_s_* are independent. To infer the influence of *x_s_* on *y*, the value of *y* was interfered with conditional probability *P(y|x_s_)*, the interfered label was denoted as 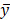, and 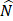
was the residuals between *y* and 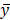. The Pearson's chi-squared test was employed to calculate P-value of the dependence of *x_s_* and 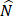 with regression. If and only if *x_s_* and 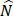 were independent, reverse was not valid, then inferred “*x_s_* is causing *y*” and was denoted as *x*
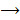
*y.*

To discover the most correlative risk factors combination of recurrence and death, the features of survival status and survival time were transformed into a 3-class label. In this label, class 1 means survival status had not occurred after 36 months, '2' means survival status had occurred within 36 months, '3' means survival status had occurred after 36 months. McDSL algorithm was employed to select the risk factors combination in each training dataset, and the significance level was set at p <0.05. The factors combination which was inferred in all training data sets, had the maximal area under the curve (AUC) and was presented to build classifiers.

### The least absolute shrinkage and selection operator algorithm

The regularized multivariate logistic regression with the LASSO penalty for discovering risk factors of 3-class label. The LASSO regularization involves a parameter λ to control the number of selected features where a larger λ retains more features. To obtain an optimal feature number and avoid over-fitting, we used 5-fold cross validation in the training cohort to choose the optimal λ. The λ value that maximizes the AUC in the training cohort was selected as the optimal regularization parameter, and the feature number was therefore determined automatically by the λ.

### Cox proportional hazards regression model

A univariate Cox analysis was firstly used to select the significant prognostic factors affecting 3-class label with backwards regression, respectively. Those factors with p <0.10 were entered into the multivariate Cox analysis. Finally, only factors with p <0.05 were deemed independent risk factors of 3-class label.

### Machine learning model training and validation

Glioma data were randomly divided into the training cohort (n = 354) and validation cohort (n = 151) with 5-fold cross-validation. The selected predictors were entered into eight machine-learning classifiers: XGBoost, Adaboost, k-Nearest Neighbor (KNN), Logistic Regression (LR), Naive Bayes (NB), Random Forest (RF), Support Vector Machine (SVM), and Back Propagation Neural Network (BPNN). Area under the curve (AUC) was used as the compared performance. The average performance of all models was obtained by bootstrapping for 1000 times. The detailed descriptions of the machine learning models are present in Document S1.

### Statistical analysis

All statistical analyses were performed by Matlab 2017b for McDSL and LASSO, and by R version 3.6.0 (http://www.Rproject.org) for eight machine learning algorithms. The R packages were used as follows: “xgboost” for XGBoost, “fitcensemble” for Adaboost, “fitcknn” for KNN, “glmfit” for LR, “fitcnb” for NB, “TreeBagger” for RF and “fitcsvm” for SVM and “nnet” for BPNN. Six performance measures of AUC, F-score, sensitivity, specificity, positive predictive value (PPV), and negative predictive value (NPV) were calculated for each model. The Youden index was used to identify the optimized threshold of predicted score that balanced sensitivity and specificity.

## Results

### Clinical characteristics of patients

Table 1 shows the comparison of patients and tumor characteristics in the training cohort and validation cohort. Among the 505 glioma cases, 175 (34.7%) were grade IV, 101 (20.0%) were grade III, and 229 (45.3%) were grade II. The mean age of patients was 48.4 ± 13.4 years, 286 (56.6%) patients were male. The 3-year PFS rate was 43.4% and 3-year OS rate was 47.9%. Of the eight possible combinations based on the presence or absence of the three tumor genetic markers, five could be used to classify most of the 471 (93.3%) gliomas: triple-positive (80, 17.0%), mutations in both TERT and IDH (14, 3.0%), mutation in IDH only (91, 19.3%), triple-negative (143, 30.4%), and mutation in TERT only (143, 30.4%).

### Performance of the machine learning models

McDSL identified four causal factors during the bootstrapping for 1000 times, including tumor location, WHO grade, histologic type, and molecular genetic group. However, LASSO and Cox analysis identified unstable number of factors of 2-17 and 1-8, respectively. Figure S1 illustrates the scale of selected features and number of each scale for McDSL, LASSO, and Cox. Figure S2 shows the variable ranking according to their importance. For McDSL, WHO grade ranked the first, followed by molecular genetic group and histologic type or tumor location; for LASSO, WHO grade ranked the first, followed by IDH mutation, and molecular group or 1p/19q codeletion; for Cox analysis, the WHO grade ranked the first, followed by radiotherapy and histologic type.

**Figure 2:**
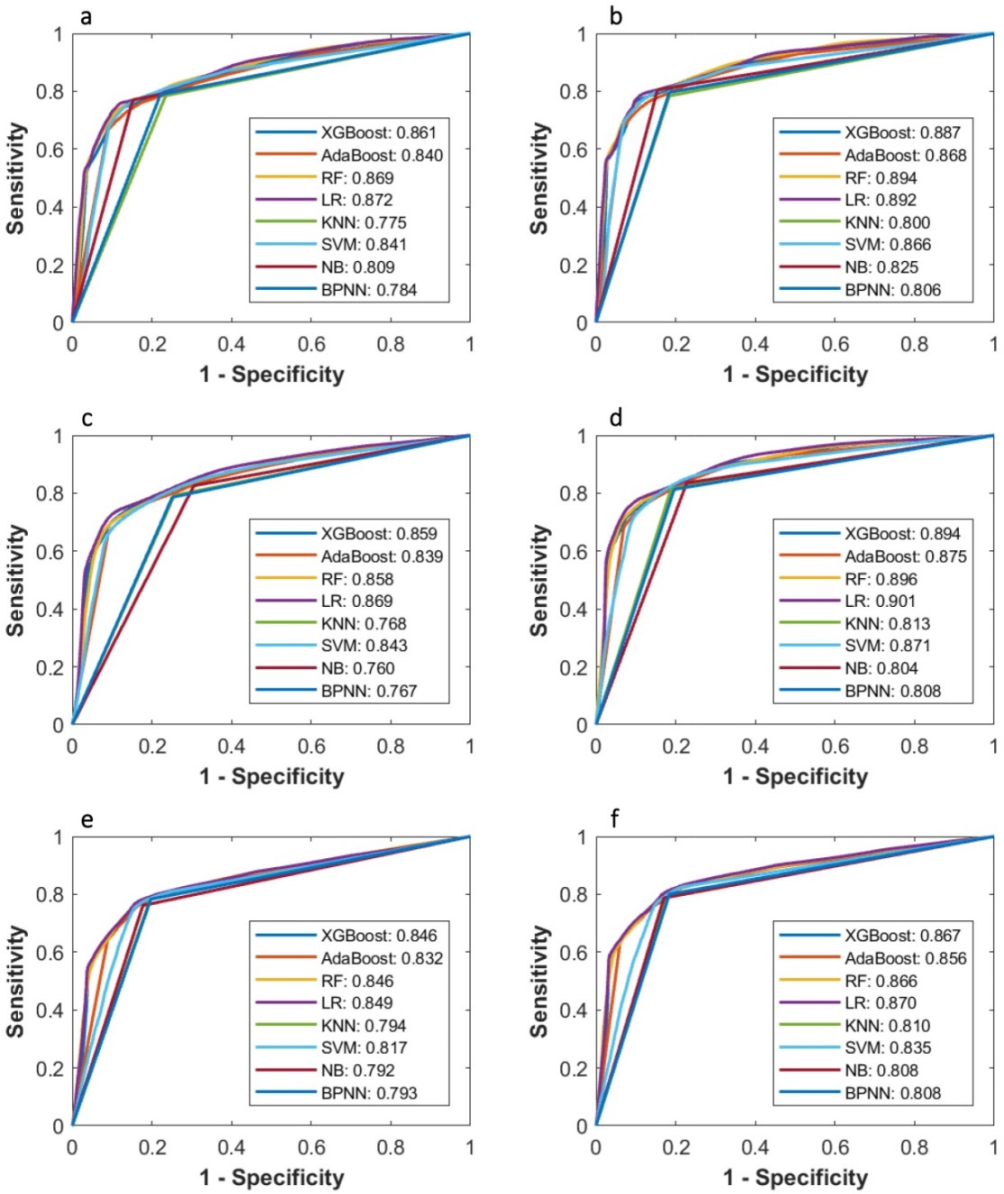
Comparison of ROC curves of eight machine learning classifiers in predicting 3-year PFS and OS probability based on Cox, LASSO, and McDSL, respectively. (a) McDSL-based 3-year PFS prediction; (b) McDSL-based 3-year OS prediction; (c) LASSO-based 3-year PFS prediction; (d) LASSO-based 3-year OS prediction; (e) Cox-based 3-year PFS prediction; and (f) Cox-based 3-year OS prediction.

Tables 2-5 summarize six prediction performance measures of classifiers based on McDSL, LASSO, Cox in predicting 3-year PFS and OS probability in the training and validation cohorts. AUCs of different models based on same feature selection method were different (Figure 2). In the validation cohort, AUCs of all models based on Cox analysis, LASSO, and McDSL in predicting 3-year PFS probability ranged from 0.792 to 0.849, 0.760 to 0.869, and 0.775 to 0.872, respectively; AUCs of all models based on Cox analysis, LASSO, and McDSL in predicting 3-year OS probability ranged from 0.808 to 0.870, 0.804 to 0.901, and 0.800 to 0.894, respectively. The LR outperformed other machine learning algorithms in predicting 3-year PFS probability based on McDSL (AUC, 0.872, 95% CI: 0.828-0.916). LR had a sensitivity of 76.2% (95% CI: 68.7%-83.7%), specificity of 87.1% (95% CI: 78.9%-95.3%), NPV of 71.0% (95% CI: 64.5%-77.5%), PPV of 90.0% (95% CI: 84.4%-95.5%), F-score of 0.824 (95% CI: 0.775-0.874). LR also outperformed other algorithms in predicting 3-year OS probability based on LASSO (AUC, 0.901, 95% CI: 0.861-0.940), which had a sensitivity of 81.0% (95% CI: 72.5%-89.5%) , specificity of 83.7% (95% CI: 72.3%-95.1%), NPV of 77.1% (95% CI: 70.0%-84.3%), PPV of 87.1% (95% CI: 79.7%-94.5%), F-score of 0.838 (95% CI: 0.792-0.884).

**Figure 3:**
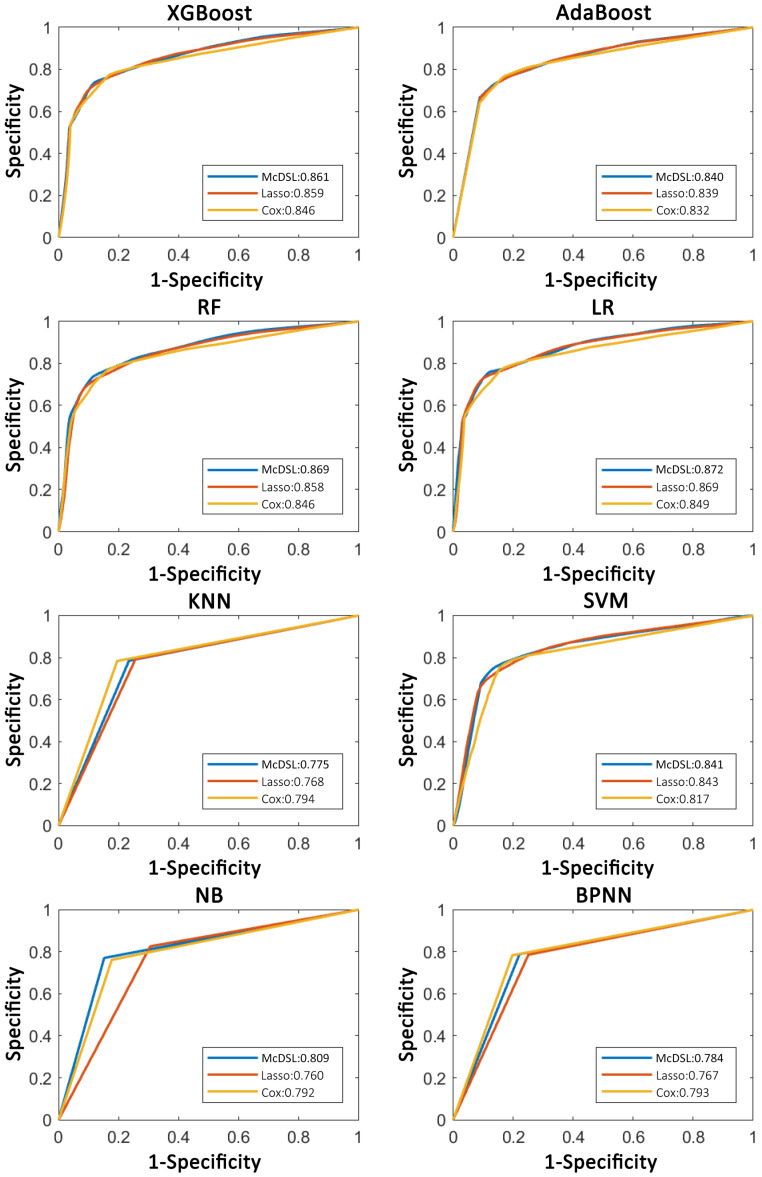
Comparison of ROC curves of each machine learning classifier based on three variable selection methods in predicting 3-year PFS probability.

**Figure 4:**
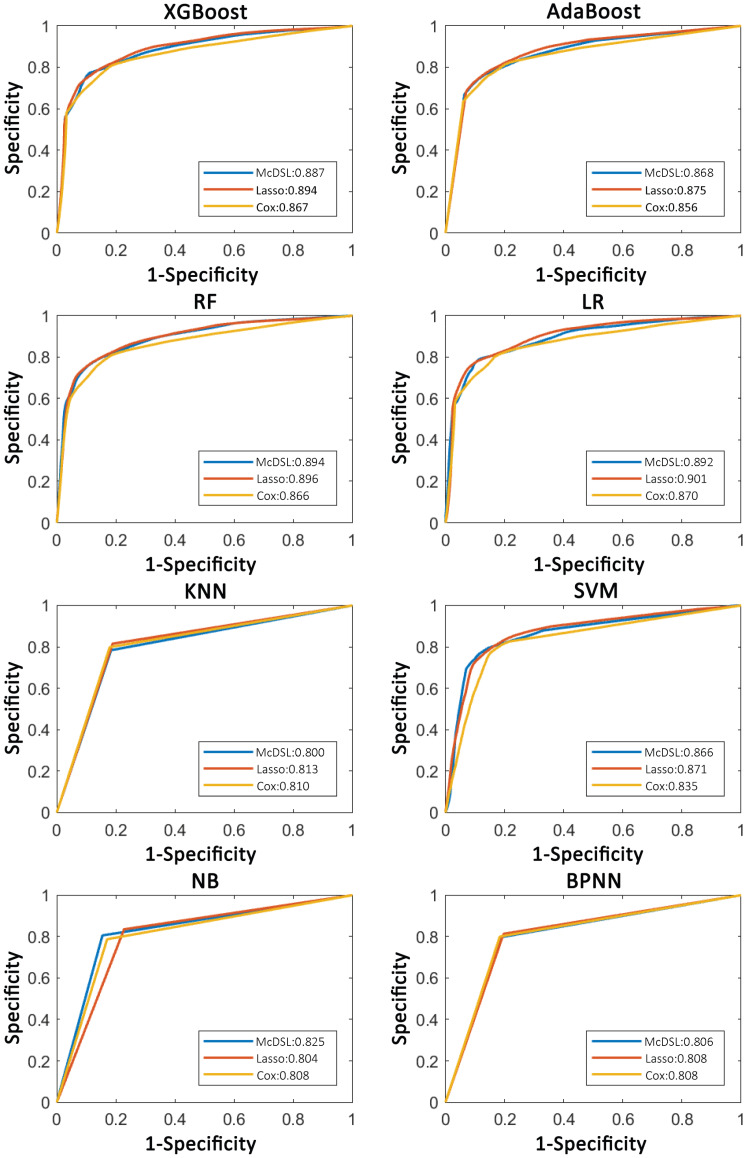
Comparison of ROC curves of each machine learning classifier based on three variable selection methods in predicting 3-year OS probability.

AUCs of most models based on McDSL, LASSO, and Cox in predicting 3-year PFS and OS probability were not significantly different (Figures 3-4). Overall, models based on McDSL and LASSO performed better than models based on Cox analysis. Specifically, AUCs of the LR based on the Cox analysis, LASSO, and McDSL in predicting 3-year PFS probability were 0.849 (95% CI: 0.800-0.898), 0.869 (95% CI: 0.823-0.915), and 0.872 (95% CI: 0.828-0.916), respectively; AUCs of the LR based on the three selection methods in predicting 3-year OS probability were 0.870 (95% CI: 0.823-0.918), 0.901 (95% CI: 0.861-0.940), and 0.892 (95% CI: 0.851-0.933), respectively.

## Discussion

Our purpose was to identify the predictors of 3-year PFS and OS probabilities in patients with gliomas based on clinical data and molecular genetic markers using three selection methods, and then develop eight machine learning models to predict 3-year PFS and OS probability. The results show most machine learning models achieved high performance in predicting 3-year PFS and OS probability, of which, LR model had the optimal predictive performance. Although the performance of machine learning models was not significantly affected by the variable selection methods, McDSL algorithm was more stable and interpretable than Cox analysis and LASSO in the feature selection process and therefore can be used as a new method to select risk factors in the future.

After an initial glioma diagnosis, standard treatment including maximal surgical resection with or without temozolomide-based chemotherapy and radiotherapy was done. Through this process, some important established prognostic factors were obtained, such as WHO grade, extent of surgical resection, and postoperative treatment 22. In this study, we found tumor location, WHO grade, histologic type, and molecular genetic group were risk factors of 3-year PFS and OS probability in patients with gliomas. Survival of gliomas is highly variable, reflecting molecular heterogeneity of the disease. Among all of the known glioma-associated molecular alterations discovered to date, the status of an IDH mutation has the largest prognostic significance. IDH mutations were noted in the vast majority of grade II and III gliomas 23, which were associated with improved survival as compared to glioblastoma (GBM). Patients with an IDH mutation gliomas had a significantly longer OS as compared with those with an IDH wildtype gliomas, with a median OS of 1.7, 6.3 and 8.0 years for patients with IDH wildtype, patients with IDH mutation only (astrocytic gliomas) and patients with IDH mutation plus 1p/19q codeletion (oligodendroglial gliomas), respectively 13. Additionally, in a recent study of grade III glioma patients treated with radiotherapy and either temozolomide or nitrosourea, IDH mutation status was found to be a significant prognostic factor for PFS (hazard ratio HR] = 0.59) and OS (HR = 0.42) 24. The 1p/19q codeletion was observed in tumors of the oligodendroglial lineage. The association between 1p/19q codeletion and prolonged OS has been observed in many previous studies. The median OS of patients with 1p/19q codeleted tumors was 11.9 years, significantly longer than that of 8.1 years for patients with 1p/19q intact tumors 25. Regardless of treatment protocol, patients with combined 1p/19q codeletion and IDH mutation had the longest PFS at 62 months as compared with 48 months for IDH mutant alone and 20 months for IDH wildtype 26. TERT promoter mutations were found in approximately 80% of IDH wildtype GBM, and in the majority of IDH mutant, 1p/19q codeleted oligodendrogliomas 9, 27, 28. In GBM, TERT promoter mutations presented worse prognosis as compared with that of patients with IDH wildtype GBM 29-31. Grade II/III gliomas with TERT promoter mutations alone harbored worse prognosis as compared with tumors with all three alteration 9. When three molecular alterations alone and their combinations (i.e., molecular group) entered the McDSL, only molecular group was selected as a cause of multiclass of survivals, suggesting the molecular group was better than single molecular genetic marker due to the molecular group provides comprehensive genetic alterations characterization of gliomas.

Machine learning has been used to capture patterns within complex data that are beyond human perception and these patterns has been adopted to make data-driven prediction 32. Overall results of machine learning models demonstrated good predictive performance for 3-year PFS and OS probability of glioma patients. In the glioma studies, machine learning algorithm has been applied directing toward discernment of MRI characteristics of tumors or a huge number of high dimensional radiomic features extracted from MRI 33. Only one non-imaging-focused study has adopted machine learning for GBM outcome prediction 34-38. However, the reliability and robustness of these models were influenced by various factors, such as interpretation of MRI images, tumor segmentation, feature and extraction, and repeatability of multiparametric features. Hence, machine learning based radiomics were insufficient for reliable clinical usage at this stage. LASSO and Cox model are commonly used to select features, but the numbers of selected features are varied after the resampling or bootstrapping. As indicated by this study, LASSO and Cox model selected a wide-range of feature number during the bootstrapping for 1000 times, in contrast, McDSL only selected four factors. Because in models constructed by LASSO and Cox model, independent variables are not all independent risk factors of survival, they are just associated with survival. However, the variables identified by McDSL have causal relationship with survival and therefore are true risk factors.

There were also some limitations should be acknowledged. Firstly, we did not consider the tumor size and intratumor features, such as necrosis size and texture features extracted from images. Previous studies showed no relationship between tumor size, necrosis size and patient survival 39-41. It is challengeable for texture features used in predicting survival outcomes due to unsolved issued of reproducibility and interpretability before application in clinical setting. Secondly, we did not perform external validation with independent datasets for generalization. Further prospective study is needed to integrate the molecular genetic markers based machine learning models into clinical practice. Thirdly, we did not consider the effects of various treatment strategies on tumor progression after standard chemoradiotherapy in OS analyses. However, this pitfall may be mitigated by performing PFS analyses.

In conclusion, our study results implied that machine learning models based on clinical profiles could obtain high performance in predicting 3-year PFS and OS probability of patients with gliomas. We demonstrated the importance of diverse sources of features in predicting survival outcomes of gliomas. The predictive model presented in this study is a preliminary step in a long-term plan of developing personalized treatment plans for glioma patients.

## Supplementary Material

Supplementary materials including figures.

## Figures and Tables

**Table 1:** Comparison of patient and tumor characteristics between the training cohort and validation cohort

Characteristics	Training cohort (n = 354)	Validation cohort (n = 151)	P value
**Age at initial diagnosis (years)**			0.449
≤40	100 (28)	37 (25)	
>40	254 (72)	114 (75)	
**Sex**			0.238
Male	207 (58)	79 (52)	
Female	147 (42)	72 (48)	
**KPS on admission**			0.809
<80	114 (32)	51 (34)	
≥80	240 (68)	100 (66)	
**Tumor location**			0.090
Frontal lobe	139 (39)	57 (38)	
Temporal lobe	66 (19)	30 (20)	
Parietal lobe	13 (4)	12 (8)	
Occipital lobe	9 (3)	2 (1)	
Insular lobe	9 (3)	9 (6)	
Multicenter	118 (32)	41 (27)	
WHO grade			0.314
II	153 (43)	76 (50)	
III	75 (21)	26 (17)	
IV	126 (36)	49 (33)	
**Histologic type**			0.009
Astrocytoma	81 (22)	54 (36)	
Oligoastrocytoma/Oligodendroglioma	147 (42)	48 (32)	
Glioblastoma	126 (36)	49 (32)	
**Extent of surgical resection**			1.000
Gross total resection	273 (77)	117 (77)	
Subtotal resection	81 (23)	34 (23)	
**Radiotherapy**			0.032
Yes	260 (73)	125 (83)	
No	94 (27)	26 (17)	
**Chemotherapy**			0.579
Temozolomide	103 (29)	48 (32)	
Nimustine/Fotemustine	100 (28)	36 (24)	
None	151 (43)	67 (44)	
**IDH1 mutation**			0.690
Yes	130 (37)	59 (39)	
No	224 (63)	92 (61)	
**IDH2 mutation**			0.888
Yes	7 (2)	4 (3)	
No	347 (98)	147 (97)	
***IDH* mutations**			0.500
Yes	137 (39)	64 (42)	
No	217 (61)	87 (58)	
***TERT* mutation**			0.019
Yes	185 (52)	61 (40)	
No	169 (48)	90 (60)	
**1p deletion**			0.514
Yes	87 (25)	42 (28)	
No	267 (75)	109 (72)	
**19q deletion**			1.000
Yes	102 (29)	43 (28)	
No	252 (71)	108 (72)	
**1p/19q codeletion**			0.924
Yes	79 (22)	35 (23)	
No	275 (78)	116 (77)	
**Molecular group**			0.315
Triple-positive	56 (16)	24 (16)	
Mutations in both *TERT* and *IDH*	11 (3)	3 (2)	
Mutation in *IDH* only	61 (17)	30 (20)	
Mutation in *TERT* only	110 (32)	33 (22)	
**Triple-negative**	93 (26)	50 (33)	
Others	23 (6)	11 (7)	
Median PFS (months)	23.5	24.8	0.852
Median OS (months)	31.1	28.4	0.726

Note: Numbers in parenthesis are percentage; SD, standard deviation; KPS, Karnofsky Performance Status; WHO, World Health Organization; *IDH*, isocitrate dehydrogenase; *TERT*, telomerase reverse transcriptase; PFS, progression free survival; OS, overall survival.

**Table 2:** The performance of eight machine learning classifiers based on McDSL, LASSO, and Cox analysis in predicting 3-year PFS probability in the training cohort

	AUC	F-score	PPV	NPV	Specificity	Sensitivity
**McDSL-based classifiers**						
XGBoost	0.855 (0.774-0.937)	0.810 (0.701-0.919)	84.6 (70.8-98.3)	70.8 (54.9-86.8)	78.0 (58.8-97.2)	78.4 (62.6-94.3)
AdaBoost	0.837 (0.748-0.926)	0.811 (0.730-0.892)	83.0 (70.2-95.8)	70.9 (54.3-87.5)	74.8 (54.4-95.2)	79.8 (67.6-92.0)
RF	0.868 (0.789-0.947)	0.822 (0.737-0.906)	86.5 (74.9-98.2)	71.7 (56.3-87.1)	81.3 (64.7-97.9)	78.7 (65.8-91.7)
LR	0.871 (0.793-0.949)	0.820 (0.737-0.903)	86.8 (75.7-97.9)	71.2 (56.2-86.2)	81.6 (65.7-97.5)	78.2 (65.5-90.9)
KNN	0.773 (0.674-0.871)	0.805 (0.716-0.893)	83.4 (71.2-95.5)	69.9 (53.8-86.0)	76.3 (59.0-93.5)	78.2 (65.3-91.1)
SVM	0.843 (0.751-0.936)	0.820 (0.736-0.905)	87.0 (75.5-98.6)	71.3 (56.3-86.3)	81.9 (64.8-99.1)	78.1 (65.0-91.2)
NB	0.813 (0.728-0.897)	0.824 (0.739-0.908)	89.2 (78.2-100)	71.2 (56.3-86.0)	85.5 (69.4-100)	77.0 (63.6-90.4)
BPNN	0.779 (0.682-0.876)	0.811 (0.729-0.894)	84.0 (71.4-96.7)	70.9 (55.9-86.0)	76.8 (57.1-96.6)	79.0 (66.1-91.9)
**LASSO-based classifiers**						
XGBoost	0.861 (0.779-0.943)	0.819 (0.732-0.905)	84.7 (72.9-96.6)	71.9 (56.0-87.8)	78.0 (60.2-95.8)	79.7 (66.4-93.0)
AdaBoost	0.841 (0.753-0.929)	0.813 (0.727-0.899)	82.8 (70.0-95.6)	71.7 (55.4-88.0)	74.3 (54.4-94.3)	80.5 (67.4-93.6)
RF	0.862 (0.780-0.944)	0.820 (0.736-0.904)	83.2 (71.5-95.0)	72.7 (56.9-88.4)	75.0 (57.6-92.5)	81.3 (69.1-93.4)
LR	0.875 (0.796-0.954)	0.822 (0.736-0.909)	85.7 (73.8-97.7)	72.1 (56.3-87.9)	79.8 (62.9-96.7)	79.5 (66.6-92.5)
KNN	0.768 (0.670-0.866)	0.806 (0.719-0.893)	82.5 (70.1-94.8)	70.5 (54.6-86.4)	74.2 (55.9-92.5)	79.4 (66.7-92.1)
SVM	0.847 (0.754-0.941)	0.816 (0.730-0.902)	84.0 (71.7-96.3)	71.7 (55.5-87.9)	76.8 (58.5-95.0)	79.9 (66.9-92.9)
NB	0.760 (0.637-0.883)	0.817 (0.734-0.901)	80.5 (66.3-94.7)	74.2 (56.4-92.0)	68.1 (39.2-97.1)	83.9 (70.4-97.3)
BPNN	0.766 (0.660-0.871)	0.803 (0.714-0.893)	82.6 (68.8-96.4)	70.2 (53.6-86.8)	74.2 (51.8-96.5)	79.0 (64.7-93.3)
**Cox-based classifiers**						
XGBoost	0.848 (0.764-0.932)	0.821 (0.737-0.904)	86.5 (75.4-97.5)	71.4 (56.7-86.1)	81.2 (65.2-97.1)	78.5 (66.1-90.9)
AdaBoost	0.835 (0.745-0.925)	0.818 (0.730-0.906)	85.6 (73.7-97.6)	71.4 (56.4-86.4)	79.6 (61.4-97.9)	78.8 (65.7-91.9)
RF	0.851 (0.764-0.938)	0.821 (0.735-0.906)	85.7 (73.6-97.8)	71.8 (56.7-87.0)	79.8 (61.3-98.2)	79.3 (66.7-91.8)
LR	0.855 (0.772-0.939)	0.824 (0.741-0.908)	87.1 (76.6-97.5)	71.7 (57.2-86.2)	82.2 (67.7-96.7)	78.6 (66.9-90.3)
KNN	0.794 (0.703-0.886)	0.818 (0.733-0.902)	86.0 (74.9-97.1)	71.0 (56.0-86.1)	80.5 (64.8-96.3)	78.3 (66.1-90.5)
SVM	0.822 (0.716-0.927)	0.820 (0.735-0.905)	86.3 (75.3-97.4)	71.4 (56.6-86.1)	81.0 (65.3-96.8)	78.5 (66.2-90.7)
NB	0.793 (0.695-0.890)	0.809 (0.717-0.901)	87.0 (75.3-98.7)	69.6 (54.3-84.9)	82.4 (64.4-100)	76.1 (62.2-90.0)
BPNN	0.794 (0.702-0.887)	0.818 (0.732-0.904)	86.0 (74.8-97.2)	71.1 (56.1-86.2)	80.5 (64.3-96.6)	78.4 (65.8-91.0)

Note: McDSL: multi-causes discovering with structure learning; AUC: area under the curve; PPV: positive predictive value; NPV: negative predictive value; KNN: k-Nearest Neighbor; LR: Logistic Regression; NB: Naive Bayes; RF: Random Forest; SVM: Support Vector Machine; BPNN: Back Propagation Neural Network.

**Table 3:** The performance of eight machine learning classifiers based on McDSL, LASSO, and Cox analysis in predicting 3-year OS probability in the training cohort

	AUC	F-score	PPV	NPV	specificity	sensitivity
**McDSL-based classifiers**						
XGBoost	0.883 (0.810-0.957)	0.825 (0.725-0.925)	85.3 (72.3-98.3)	76.5 (61.8-91.2)	81.3 (64.3-98.3)	80.6 (65.3-95.8)
AdaBoost	0.868 (0.784-0.951)	0.823 (0.736-0.910)	84.4 (71.6-97.2)	76.1 (60.9-91.4)	80.0 (62.2-97.7)	80.9 (68.0-93.7)
RF	0.895 (0.823-0.966)	0.834 (0.750-0.918)	87.6 (75.9-99.4)	76.6 (62.6-90.6)	84.9 (69.5-100)	80.0 (66.8-93.2)
LR	0.893 (0.821-0.965)	0.837 (0.756-0.918)	87.6 (76.6-98.7)	77.0 (63.8-90.2)	84.8 (70.8-98.9)	80.5 (68.1-93.0)
KNN	0.801 (0.707-0.895)	0.815 (0.722-0.908)	84.6 (72.1-97.2)	74.7 (60.2-89.2)	81.1 (65.6-96.6)	79.0 (66.0-92.1)
SVM	0.870 (0.785-0.954)	0.834 (0.750-0.917)	87.5 (76.0-98.9)	76.6 (63.2-90.0)	84.7 (70.2-99.2)	80.1 (67.3-92.9)
NB	0.830 (0.749-0.910)	0.838 (0.752-0.924)	88.2 (76.6-99.7)	77.3 (63.0-91.7)	85.4 (70.0-100)	80.5 (66.0-95.0)
BPNN	0.804 (0.715-0.894)	0.820 (0.736-0.904)	84.9 (72.1-97.8)	75.6 (62.2-89.0)	81.0 (63.5-98.6)	79.8 (67.0-92.7)
**LASSO-based classifiers**						
XGBoost	0.897 (0.827-0.967)	0.837 (0.752-0.923)	85.9 (73.9-97.9)	78.1 (63.4-92.7)	82.1 (66.2-97.9)	82.2 (68.9-95.6)
AdaBoost	0.880 (0.803-0.957)	0.835 (0.748-0.921)	84.9 (72.4-97.5)	78.2 (63.2-93.1)	80.4 (63.2-97.7)	82.6 (69.2-96.1)
RF	0.903 (0.834-0.971)	0.842 (0.760-0.925)	84.8 (72.9-96.6)	79.5 (65.3-93.7)	80.0 (64.1-95.9)	84.2 (72.3-96.1)
LR	0.907 (0.843-0.972)	0.841 (0.760-0.921)	86.4 (74.4-98.3)	78.3 (64.4-92.2)	82.7 (67.2-98.3)	82.4 (70.0-94.8)
KNN	0.818 (0.729-0.908)	0.835 (0.748-0.921)	85.6 (73.3-97.8)	77.7 (63.1-92.2)	81.7 (65.8-97.6)	82.0 (69.2-94.8)
SVM	0.878 (0.783-0.973)	0.840 (0.755-0.925)	85.5 (73.4-97.5)	78.7 (63.5-93.9)	81.3 (65.3-97.4)	83.1 (69.8-96.3)
NB	0.809 (0.701-0.917)	0.836 (0.750-0.922)	83.6 (69.7-97.5)	79.4 (64.5-94.3)	77.4 (53.6-100)	84.4 (71.4-97.3)
BPNN	0.811 (0.718-0.905)	0.829 (0.742-0.917)	84.9 (71.8-98.0)	77.2 (62.2-92.2)	80.6 (62.3-98.8)	81.7 (68.1-95.2)
**Cox-based classifiers**						
XGBoost	0.870 (0.789-0.952)	0.827 (0.738-0.916)	85.9 (74.1-97.7)	76.2 (62.1-90.4)	82.5 (67.6-97.4)	80.2 (67.0-93.5)
AdaBoost	0.861 (0.778-0.944)	0.820 (0.727-0.914)	85.6 (72.8-98.3)	75.5 (60.4-90.7)	82.0 (65.1-99.0)	79.5 (64.5-94.5)
RF	0.872 (0.790-0.954)	0.828 (0.742-0.914)	85.6 (73.9-97.3)	76.5 (62.2-90.7)	82.1 (67.2-96.9)	80.7 (68.1-93.3)
LR	0.876 (0.795-0.957)	0.833 (0.749-0.917)	86.2 (74.9-97.5)	76.9 (63.4-90.4)	82.9 (68.7-97.1)	81.0 (69.0-93.0)
KNN	0.812 (0.725-0.898)	0.824 (0.738-0.911)	85.9 (74.5-97.2)	75.7 (61.7-89.7)	82.6 (68.2-97.0)	79.7 (66.9-92.6)
SVM	0.842 (0.738-0.945)	0.827 (0.741-0.913)	85.5 (74.3-96.8)	76.2 (62.3-90.2)	81.9 (67.4-96.5)	80.4 (67.9-93.0)
NB	0.810 (0.718-0.902)	0.820 (0.727-0.913)	86.3 (74.3-98.3)	75.0 (60.8-89.2)	83.4 (67.7-99.0)	78.7 (65.1-92.2)
BPNN	0.809 (0.720-0.898)	0.823 (0.734-0.912)	85.5 (73.8-97.2)	75.8 (61.5-90.0)	82.0 (67.0-97.0)	79.9 (66.8-93.0)

Note: McDSL: multi-causes discovering with structure learning; AUC: area under the curve; PPV: positive predictive value; NPV: negative predictive value; KNN: k-Nearest Neighbor; LR: Logistic Regression; NB: Naive Bayes; RF: Random Forest; SVM: Support Vector Machine; BPNN: Back Propagation Neural Network.

**Table 4:** The performance of eight machine learning classifiers based on McDSL, LASSO, and Cox analysis in predicting 3-year PFS probability in the validation cohort

	AUC	F-score	PPV	NPV	Specificity	Sensitivity
**McDSL-based classifiers**						
XGBoost	0.861 (0.814-0.909)	0.816 (0.768-0.864)	85.0 (77.3-92.6)	71.4 (63.9-78.9)	78.6 (64.7-92.6)	78.8 (69.7-87.8)
AdaBoost	0.840 (0.787-0.894)	0.812 (0.762-0.861)	82.8 (74.8-90.8)	71.5 (63.2-79.7)	74.5 (59.0-90.0)	79.9 (70.7-89.1)
RF	0.869 (0.824-0.915)	0.821 (0.772-0.870)	86.2 (78.1-94.4)	71.7 (64.6-78.9)	80.7 (66.2-95.2)	78.7 (70.4-86.9)
LR	0.872 (0.828-0.916)	0.824 (0.775-0.874)	90.0 (84.4-95.5)	71.0 (64.5-77.5)	87.1 (78.9-95.3)	76.2 (68.7-83.7)
KNN	0.775 (0.717-0.833)	0.808 (0.758-0.859)	83.6 (77.2-89.9)	70.5 (62.7-78.4)	76.5 (64.9-88.1)	78.5 (69.8-87.1)
SVM	0.841 (0.785-0.898)	0.822 (0.773-0.870)	87.0 (79.4-94.7)	71.5 (64.5-78.5)	82.1 (68.6-95.7)	78.1 (69.8-86.3)
NB	0.809 (0.742-0.876)	0.823 (0.775-0.872)	88.8 (79.4-98.2)	71.3 (64.7-77.9)	84.8 (68.4-100)	77.0 (68.2-85.9)
BPNN	0.784 (0.721-0.846)	0.814 (0.764-0.865)	84.5 (76.9-92.1)	71.3 (63.3-79.3)	77.9 (64.1-91.6)	78.9 (69.9-87.9)
**LASSO-based classifiers**						
XGBoost	0.859 (0.812-0.907)	0.818 (0.770-0.866)	84.7 (77.4-92.0)	71.9 (63.9-79.9)	78.0 (64.7-91.4)	79.4 (70.2-88.7)
AdaBoost	0.839 (0.786-0.893)	0.813 (0.763-0.863)	82.6 (74.7-90.5)	71.7 (63.3-80.1)	74.1 (58.8-89.4)	80.3 (71.2-89.4)
RF	0.858 (0.810-0.907)	0.820 (0.774-0.866)	83.0 (76.7-89.3)	72.8 (65.0-80.6)	74.7 (62.8-86.7)	81.2 (73.2-89.3)
LR	0.869 (0.823-0.915)	0.818 (0.769-0.868)	88.0 (80.3-95.7)	70.8 (63.6-78.0)	83.9 (71.1-96.6)	76.7 (67.7-85.8)
KNN	0.768 (0.706-0.830)	0.807 (0.759-0.855)	82.5 (75.4-89.6)	70.6 (63.0-78.3)	74.4 (61.1-87.7)	79.2 (71.0-87.5)
SVM	0.843 (0.786-0.900)	0.817 (0.769-0.864)	84.3 (77.0-91.6)	71.7 (64.1-79.4)	77.3 (63.7-90.9)	79.5 (70.6-88.4)
NB	0.760 (0.678-0.843)	0.814 (0.768-0.860)	80.6 (71.9-89.3)	73.0 (64.1-82.0)	69.4 (48.7-90.2)	82.6 (73.5-91.7)
BPNN	0.767 (0.694-0.841)	0.805 (0.753-0.856)	82.9 (73.4-92.4)	70.3 (62.0-78.5)	74.9 (56.4-93.4)	78.6 (68.4-88.8)
**Cox-based classifiers**						
XGBoost	0.846 (0.795-0.896)	0.821 (0.770-0.872)	86.3 (79.6-93.0)	71.7 (64.2-79.1)	81.0 (69.5-92.5)	78.5 (70.3-86.7)
AdaBoost	0.832 (0.776-0.888)	0.818 (0.763-0.873)	85.3 (77.2-93.3)	71.6 (63.8-79.4)	79.1 (64.2-93.9)	78.9 (70.0-87.8)
RF	0.846 (0.796-0.896)	0.821 (0.771-0.871)	85.5 (78.2-92.9)	71.9 (64.4-79.5)	79.5 (65.5-93.5)	79.1 (70.7-87.6)
LR	0.849 (0.800-0.898)	0.804 (0.723-0.885)	88.6 (81.6-95.6)	69.2 (59.4-79.0)	85.2 (73.9-96.6)	74.1 (59.8-88.5)
KNN	0.794 (0.734-0.854)	0.819 (0.767-0.871)	85.9 (79.4-92.4)	71.4 (64.0-78.8)	80.4 (69.4-91.4)	78.4 (70.5-86.2)
SVM	0.817 (0.746-0.889)	0.820 (0.769-0.871)	86.2 (79.9-92.6)	71.5 (64.2-78.8)	81.0 (70.3-91.7)	78.3 (70.2-86.4)
NB	0.792 (0.724-0.860)	0.810 (0.750-0.870)	86.8 (79.2-94.5)	69.9 (61.7-78.1)	82.3 (68.1-96.4)	76.1 (66.0-86.2)
BPNN	0.793 (0.730-0.856)	0.818 (0.764-0.871)	85.8 (78.9-92.6)	71.3 (63.7-78.9)	80.3 (68.7-91.8)	78.3 (70.2-86.4)

Note: McDSL: multi-causes discovering with structure learning; AUC: area under the curve; PPV: positive predictive value; NPV: negative predictive value; KNN: k-Nearest Neighbor; LR: Logistic Regression; NB: Naive Bayes; RF: Random Forest; SVM: Support Vector Machine; BPNN: Back Propagation Neural Network.

**Table 5:** The performance of eight machine learning classifiers based on McDSL, LASSO, and Cox analysis in predicting 3-year OS probability in the validation cohort

	AUC	F-score	PPV	NPV	Specificity	Sensitivity
**McDSL-based classifiers**						
XGBoost	0.887 (0.844-0.930)	0.829 (0.782-0.877)	85.7 (77.3-94.2)	76.4 (69.3-83.5)	81.8 (68.4-95.2)	80.7 (71.9-89.4)
AdaBoost	0.868 (0.820-0.917)	0.823 (0.774-0.872)	84.3 (75.9-92.8)	75.9 (68.5-83.3)	79.7 (65.8-93.7)	80.6 (71.8-89.5)
RF	0.894 (0.854-0.935)	0.834 (0.786-0.882)	87.7 (78.6-96.8)	76.2 (69.4-83.0)	84.7 (70.3-99.0)	79.8 (71.3-88.3)
LR	0.892 (0.851-0.933)	0.841 (0.795-0.887)	89.2 (83.0-95.4)	76.5 (70.4-82.6)	87.0 (78.1-96.0)	79.7 (72.6-86.8)
KNN	0.800 (0.745-0.855)	0.815 (0.764-0.866)	85.2 (77.9-92.4)	74.2 (67.2-81.3)	81.6 (70.5-92.7)	78.4 (69.8-86.9)
SVM	0.866 (0.815-0.917)	0.834 (0.786-0.882)	87.6 (79.6-95.6)	76.2 (69.7-82.7)	84.7 (72.1-97.2)	79.9 (71.9-87.8)
NB	0.825 (0.767-0.884)	0.838 (0.790-0.886)	87.8 (78.2-97.4)	77.1 (68.9-85.2)	84.6 (68.6-100)	80.5 (70.1-91.0)
BPNN	0.806 (0.746-0.867)	0.823 (0.772-0.874)	85.4 (77.1-93.7)	75.3 (68.2-82.5)	81.6 (68.6-94.7)	79.6 (71.0-88.2)
**LASSO-based classifiers**						
XGBoost	0.894 (0.851-0.936)	0.836 (0.786-0.885)	85.6 (78.2-93.1)	77.6 (69.4-85.8)	81.4 (69.4-93.5)	81.9 (72.4-91.4)
AdaBoost	0.875 (0.824-0.926)	0.833 (0.780-0.885)	84.6 (76.0-93.3)	77.7 (69.0-86.3)	79.7 (65.3-94.1)	82.4 (72.5-92.2)
RF	0.896 (0.856-0.936)	0.840 (0.794-0.886)	84.3 (77.6-91.0)	79.0 (71.4-86.6)	79.0 (67.8-90.3)	83.9 (75.8-91.9)
LR	0.901 (0.861-0.940)	0.838 (0.792-0.884)	87.1 (79.7-94.5)	77.1 (70.0-84.3)	837 (72.3-95.1)	81.0 (72.5-89.5)
KNN	0.813 (0.756-0.870)	0.833 (0.783-0.882)	85.3 (77.9-92.7)	77.1 (69.5-84.6)	81.1 (69.5-92.7)	81.5 (73.0-90.0)
SVM	0.871 (0.805-0.936)	0.838 (0.787-0.889)	85.4 (78.2-92.5)	78.1 (69.8-86.4)	80.9 (69.2-92.7)	82.6 (73.3-91.9)
NB	0.804 (0.726-0.881)	0.832 (0.781-0.883)	83.4 (73.7-93.1)	78.4 (69.9-86.9)	77.2 (58.3-96.2)	83.5 (74.4-92.7)
BPNN	0.808 (0.741-0.876)	0.829 (0.775-0.883)	85.0 (75.7-94.2)	-	80.4 (64.7-96.0)	81.3 (71.4-91.2)
**Cox-based classifiers**						
XGBoost	0.867 (0.819-0.915)	0.828 (0.776-0.881)	85.6 (78.8-92.3)	76.3 (68.6-84.0)	81.8 (71.4-92.1)	80.5 (71.5-89.5)
AdaBoost	0.856 (0.805-0.907)	0.821 (0.758-0.884)	85.5 (77.7-93.3)	75.4 (66.3-84.4)	81.7 (69.5-93.9)	79.4 (68.0-90.8)
RF	0.866 (0.816-0.916)	0.829 (0.780-0.878)	85.4 (79.0-91.9)	76.4 (69.1-83.7)	81.6 (71.6-91.6)	80.7 (72.2-89.2)
LR	0.870 (0.823-0.918)	0.809 (0.730-0.887)	88.6 (80.2-96.9)	73.0 (62.9-83.1)	86.6 (74.2-98.9)	75.1 (59.7-90.4)
KNN	0.810 (0.757-0.862)	0.826 (0.774-0.877)	85.7 (79.3-92.0)	75.7 (68.4-83.0)	82.1 (72.6-91.6)	79.9 (71.3-88.4)
SVM	0.835 (0.760-0.910)	0.827 (0.777-0.877)	85.4 (78.9-91.9)	76.0 (68.9-83.1)	81.6 (71.7-91.5)	80.4 (72.2-88.5)
NB	0.808 (0.747-0.870)	0.821 (0.762-0.880)	86.1 (78.9-93.4)	74.9 (66.8-83.0)	83.0 (71.6-94.3)	78.7 (69.0-88.4)
BPNN	0.808 (0.752-0.864)	0.825 (0.771-0.879)	85.4 (78.6-92.1)	75.7 (68.2-83.3)	81.6 (71.4-91.8)	80.0 (71.3-88.7)

Note: McDSL: multi-causes discovering with structure learning; AUC: area under the curve; PPV: positive predictive value; NPV: negative predictive value; KNN: k-Nearest Neighbor; LR: Logistic Regression; NB: Naive Bayes; RF: Random Forest; SVM: Support Vector Machine; BPNN: Back Propagation Neural Network.

## References

[1] Molinaro AM, Taylor JW, Wiencke JK (2019). Genetic and molecular epidemiology of adult diffuse glioma. Nat Rev Neurol.

[2] Schwartzbaum JA, Fisher JL, Aldape KD (2006). Epidemiology and molecular pathology of glioma. Nat Clin Pract Neurol.

[3] Ferlay J, Colombet M, Soerjomataram I (2019). Estimating the global cancer incidence and mortality in 2018: GLOBOCAN sources and methods. Int J Cancer.

[4] Pope WB, Kim HJ, Huo J (2009). Recurrent glioblastoma multiforme: ADC histogram analysis predicts response to bevacizumab treatment. Radiology.

[5] Aquilanti E, Miller J, Santagata S (2018). Updates in prognostic markers for gliomas. Neuro Oncol.

[6] Prestwich RJ, Sivapalasunrtharam A, Johnston C (2005). Survival in high-grade glioma: a study of survival in patients unfit for or declining radiotherapy. Clin Oncol (R Coll Radiol).

[7] Miller JJ, Loebel F, Juratli TA (2019). Accelerated progression of IDH mutant glioma after first recurrence. Neuro Oncol.

[8] Claus EB, Walsh KM, Wiencke JK (2015). Survival and low-grade glioma: the emergence of genetic information. Neurosurg Focus.

[9] Eckel-Passow JE, Lachance DH, Molinaro AM (2015). Glioma Groups Based on 1p/19q, IDH, and TERT Promoter Mutations in Tumors. N Engl J Med.

[10] Louis DN, Perry A, Reifenberger G (2016). The 2016 World Health Organization Classification of Tumors of the Central Nervous System: a summary. Acta Neuropathol.

[11] Reifenberger G, Wirsching HG, Knobbe-Thomsen CB (2017). Advances in the molecular genetics of gliomas - implications for classification and therapy. Nat Rev Clin Oncol.

[12] van den Bent MJ, Weller M, Wen PY (2017). A clinical perspective on the 2016 WHO brain tumor classification and routine molecular diagnostics. Neuro Oncol.

[13] Brat DJ, Verhaak RG, Aldape KD (2015). Comprehensive, Integrative Genomic Analysis of Diffuse Lower-Grade Gliomas. N Engl J Med.

[14] Ceccarelli M, Barthel FP, Malta TM (2016). Molecular Profiling Reveals Biologically Discrete Subsets and Pathways of Progression in Diffuse Glioma. Cell.

[15] Zhang Z, Chan AK, Ding X (2015). TERT promoter mutations contribute to IDH mutations in predicting differential responses to adjuvant therapies in WHO grade II and III diffuse gliomas. Oncotarget.

[16] Yao Y, Chan AK, Qin ZY (2013). Mutation analysis of IDH1 in paired gliomas revealed IDH1 mutation was not associated with malignant progression but predicted longer survival. Plos One.

[17] Woehrer A, Sander P, Haberler C (2011). FISH-based detection of 1p 19q codeletion in oligodendroglial tumors: procedures and protocols for neuropathological practice - a publication under the auspices of the Research Committee of the European Confederation of Neuropathological Societies (Euro-CNS). Clin Neuropathol.

[18] Chan AK, Yao Y, Zhang Z (2015). TERT promoter mutations contribute to subset prognostication of lower-grade gliomas. Modern pathology: an official journal of the United States and Canadian Academy of Pathology, Inc.

[19] Chen W, Hu Y, Zhang X (2018). Causal risk factor discovery for severe acute kidney injury using electronic health records. BMC Med Inform Decis Mak.

[20] Chen W, Hao Z, Cai R (2016). Multiple-cause discovery combined with structure learning for high-dimensional discrete data and application to stock prediction. Soft Comput.

[21] Peters J, Janzing D, Scholkopf B (2011). Causal Inference on Discrete Data Using Additive Noise Models. Ieee T Pattern Anal.

[22] Bae S, Choi YS, Ahn SS (2018). Radiomic MRI Phenotyping of Glioblastoma: Improving Survival Prediction. Radiology.

[23] Yan H, Parsons DW, Jin G (2009). IDH1 and IDH2 mutations in gliomas. N Engl J Med.

[24] Chang S, Zhang P, Cairncross JG (2017). Phase III randomized study of radiation and temozolomide versus radiation and nitrosourea therapy for anaplastic astrocytoma: results of NRG Oncology RTOG 9813. Neuro Oncol.

[25] Jenkins RB, Blair H, Ballman KV (2006). A t(1;19)(q10;p10) mediates the combined deletions of 1p and 19q and predicts a better prognosis of patients with oligodendroglioma. Cancer Res.

[26] Baumert BG, Hegi ME, van den Bent MJ (2016). Temozolomide chemotherapy versus radiotherapy in high-risk low-grade glioma (EORTC 22033-26033): a randomised, open-label, phase 3 intergroup study. Lancet Oncol.

[27] Killela PJ, Reitman ZJ, Jiao Y (2013). TERT promoter mutations occur frequently in gliomas and a subset of tumors derived from cells with low rates of self-renewal. Proc Natl Acad Sci U S A.

[28] Brennan CW, Verhaak RG, McKenna A (2013). The somatic genomic landscape of glioblastoma. Cell.

[29] Chamberlain MC, Sanson M (2015). Combined analysis of TERT, EGFR, and IDH status defines distinct prognostic glioblastoma classes. Neurology.

[30] Simon M, Hosen I, Gousias K (2015). TERT promoter mutations: a novel independent prognostic factor in primary glioblastomas. Neuro Oncol.

[31] Spiegl-Kreinecker S, Lotsch D, Ghanim B (2015). Prognostic quality of activating TERT promoter mutations in glioblastoma: interaction with the rs2853669 polymorphism and patient age at diagnosis. Neuro Oncol.

[32] Lotan E, Jain R, Razavian N (2019). State of the Art: Machine Learning Applications in Glioma Imaging. AJR Am J Roentgenol.

[33] Liu X, Li Y, Qian Z (2018). A radiomic signature as a non-invasive predictor of progression-free survival in patients with lower-grade gliomas. Neuroimage Clin.

[34] Malhotra K, Navathe SB, Chau DH (2016). Constraint based temporal event sequence mining for Glioblastoma survival prediction. J Biomed Inform.

[35] Wu G, Shi Z, Chen Y (2019). A sparse representation-based radiomics for outcome prediction of higher grade gliomas. Med Phys.

[36] Kim BS, Kim ST, Kim JH (2019). Apparent Diffusion Coefficient as a Predictive Biomarker for Survival in Patients with Treatment-Naive Glioblastoma Using Quantitative Multiparametric Magnetic Resonance Profiling. World Neurosurg.

[37] Sanghani P, Ang BT, King N (2018). Overall survival prediction in glioblastoma multiforme patients from volumetric, shape and texture features using machine learning. Surg Oncol.

[38] Liu L, Zhang H, Wu J (2018). Overall survival time prediction for high-grade glioma patients based on large-scale brain functional networks. Brain Imaging Behav.

[39] Alimohammadi E, Bagheri SR, Sadeghsalehi A (2020). Prognostic factors in patients with glioblastoma multiforme: focus on the pathologic variants. Acta Neurol Belg.

[40] Muhammed Amr, Gaber Mohamed S, Elbeltagy Mohamed (2019). Risk stratification of pediatric high-grade glioma: a newly proposed prognostic score. Childs Nerv Syst.

[41] Deng L, Shen L, Shen L (2019). Prognostic value of magnetic resonance imaging features in low-grade gliomas. Biosci Rep.

