# Supplementary material for: Machine learning classifiers for predicting 3-year progression-free survival and overall survival in patients with gliomas after surgery
Source: J Cancer. 2021 Jan 15;12(6):1604–15. doi: 10.7150/jca.52183 (PMC7890310; doi:10.7150/jca.52183)

Supplementary Materials

Document S1: Method Description of Machine-Learning Algorithms

RF: Random Forests (RFs) are designed to further improve the accuracy of models by building multiple trees i.e. a Forest. To make a prediction for a new observation, each tree in the forest gives a classification (a vote) on the outcome and the forest choose the classification having the majority votes (over all trees in the forest). To build a Random Forest, each constituent tree is forced to split on only a random subset of the available independent variables from a bootstrapped sample of the data. For example, the training data for each tree is selected randomly with replacement-since each tree uses different independent variables and different training data, we generate a Forest of different trees.

RF is a modification of bagging that builds and averages many trees to obtain an approximately unbiased models to reduce the variance. RF also has the ability to avoid the high variance or bias in prediction and is very simple to be implemented efficiently and accurately. Precisely, the RF will grow *B* trees to the bootstrapped data and repeating some statistical procedure to finally return the ensemble of the grown trees. When a new observation *x* is coming, let the $\widehat{D}_{RF}(x)$ be the class prediction of the b-th tree, the

$$\hat{D}_{RF}(\mathrm{x}) = \text{majority} \quad \text{vote}\{\hat{D}^{b}{}_{RF}(\mathrm{x})\}_{b=1}^{B}$$

L²-LOG: The logistic regression can be regarded as a generalized linear model with the logit link function. It arises from the desire to model the posterior probabilities of the classes via linear functions in *x*, while at the same time ensuring that they sum to one and remain in [0, 1]. In the binary case, the model has the form:

$$\log\frac{P(y=1\,|\,x)}{P(y=0\,|\,x)} = \beta_0 + \sum_{k=1}^{p}\beta_k x_k$$

Furthermore, denote $p(x) = P(y = 1/x)$, then the L^2-Logistic regression aims to solve the following optimization problem:

$$\operatorname{argmax}\frac{1}{n}\sum_{i=1}^{n}(y_i \log p(\mathrm{x}_i) + (1-y_i)\log(1-p(\mathrm{x}_i))) - \lambda\sum_{k=1}^{p}{\beta_k}^2$$

Therefore, we obtain the desired classifier.

k-SVM: SVM can be formulated in the context of binary classification as the model that finds the decision boundary which maximizes the margin between two data classes. The Gaussian kernel is applied in the kernel-SVM, which is a very powerful tool in classification and has

great performance in different kinds of real-life applications. Precisely, considering the two class binary classification problem, the SVM aims to find a hyperplane which maximizes the margin between two classes. Generally, the classifier is assumed to be contained in a reproducing kernel Hilbert space (RKHS) induced by some kernel function *K*, denoted as *HK*. More importantly, the classifiers can be learnt from high dimensional features spaces and represent by a form of finite linear combination of the kernel functions. It is well known that any continuous function can be well approximated by the function in the RKHS if the kernel *K* is universal, for example, the Gaussian kernel. The classification problem of SVM can be formulated to solve the following optimization problem

$$\min_{f \in \mathrm{H}_K} \frac{1}{n} \sum_{i=1}^{n} (1 - \mathrm{y}_i f(\mathrm{x}_i))_+ + \lambda \|f\|_{\mathrm{H}_K}^2$$

Such a problem can be efficiently solved by transforming the objective function to its dual problem and applying the KKT condition. By the representer theorem, the solution of such a optimization problem must satisfy that

$$\hat{f}(\mathrm{x}) = \sum_{i=1}^{n} \hat{\alpha}_i K(\mathrm{x}_i, \mathrm{x})$$

Where $\hat{\alpha}_i$ is the representer coefficients.

AdaBoost: The AdaBoost combines several weak classifiers together to construct a good classifier. Precisely, supposing that G(*x*) is a classifier that predict the label of the new coming observation. Then its error rate can be computed as

$$Error = \frac{1}{n} \sum_{i=1}^{n} 1_{\{\mathrm{y}_i \neq \mathrm{G}(\mathrm{x}_i)\}}$$

We call G(*x*) is a weak classifier if its error rate is slightly better than the random guess. The AdaBoost classifier combines the performance of a sequential weak classifiers $G_1(x_i)$, ..., $G_m(x_i)$ in the sense that

$$G_{\mathrm{ad}b}(\mathrm{x}) = \mathrm{sign}(\sum_{k=1}^{m} \alpha_k G_k(\mathrm{x}))$$

where α_k is the weight for the k-th weak classifier.

k-NN: The k-NN is a instance-based learning method, where the classification function is approximated by the local data. It is very simple and flexible with widely successful applications. k-NN uses the training samples to predict a new sample by a majority vote on the outcome of the k-nearest points to the new sample. k-NN method is a very simple and efficient method for classification. Given the sample $\{(y_i, x_i)\}_{i=1}^{n}$, the k-nearest neighbor

uses those observations in the training set which are closest to *x* to predict y. In detail, by using the Euclidean distance as a metric to measure the closeness, the k-NN can be fitted that

$$\hat{y} = \frac{1}{k} \sum_{x_i \hat{I} N_k(x)} y_i$$

where *Nk* (*x*) is the neighborhood of *x* defined by the *k* closest points x_i in the training set.

NB: The NB classifier is a probabilistic based method, which applies Bayes' theorem with strong (naive) independence assumptions between the variables, and it will converge more faster than some discriminative models, for example logistic regression, and thus fewer training sample is required to achieve an accurate classification result. NB is a popularly used classification method which bases on Baye's theorem with the 'naive' assumption of independence between every pair of features. Give the pair (*y*; *x*), where *y* = {-1, 1} is the label and $x = (x_1, \ldots, x_p)^T$ denotes the variables. The Baye's theorem is formulated as

$$P(y \mid x) = \frac{P(y)P(x \mid y)}{P(x)}$$

With the naive independence assumption, the classifier is constructed:

$$\hat{y} = \arg \max_{y \in \{-1,1\}} P(y) \prod_{i=1}^{p} P(x_i \mid y)$$

Then the label can be assigned by the probability model.

XGBoost： XGBoost is an optimized distributed machine learning algorithm, which under the parallel gradient tree boosting framework (also known as GBDT, GBM). Different from GBDT, a regularized objective was added into loss function to smooth the final learnt weights to avoid over-fitting, which is formulated as

$$\mathcal{L}(\phi) = \sum_i l(\hat{y}_i, y_i) + \sum_k \Omega(f_k)$$

$$\text{where } \Omega(f) = \gamma T + \frac{1}{2}\lambda \|w\|^2$$

Here l is the loss function between the prediction $\hat{y}_i$ and the target y_i, and T is the number of leaf. Moreover, the minimizing objective function is formulated as

$$Obj = -\frac{1}{2}\sum_{j=1}^{T} \frac{G_j^2}{H_j + \lambda} + \gamma T$$

Here G_j and H_j are denoted as the sum of the first derivative and the second derivative of loss function respectively.

BPNN: A Back-Propagation neural network (BPNN) the most basic neural network that learns by constantly modifying both the connection weights between the neurons in each layer and the neuron thresholds to make the network output continuously approximate the desired output. Because a BPNN is robust and can realize any complex nonlinear mapping relation, this technique has been widely used in many fields.

References

[1] Hastie, T., R. Tibshirani, and J. Friedman. The Elements of Statistical Learning, 2nd edition. Springer, New York.2009.

[2] Zou, H. and T. Hastie. Regularization and variable selection via the elastic net. Journal of the Royal Statistical Society, Series B, 2005;67, 301–-320.

[3] Genuer, R. and Poggi, J. and Tuleau-Malot, C.VSURF: An R Package for Variable Selection Using Random Forests, The R Journal, 2015; 7, 19-33.

[4] Chen T, Guestrin C. Xgboost: A scalable tree boosting system[C]//Proceedings of the 22nd acm sigkdd international conference on knowledge discovery and data mining. ACM, 2016: 785-794.

[5] Cao J, Cui H, Shi H, Jiao L. Big Data: A Parallel Particle Swarm Optimization-Back-Propagation Neural Network Algorithm Based on MapReduce. *PLoS One*. 2016 Jun 15;11(6):e0157551. doi: 10.1371/journal.pone.0157551.

Figure S1: The scale of selected features and number of each scale for McDSL, LASSO, and Cox model

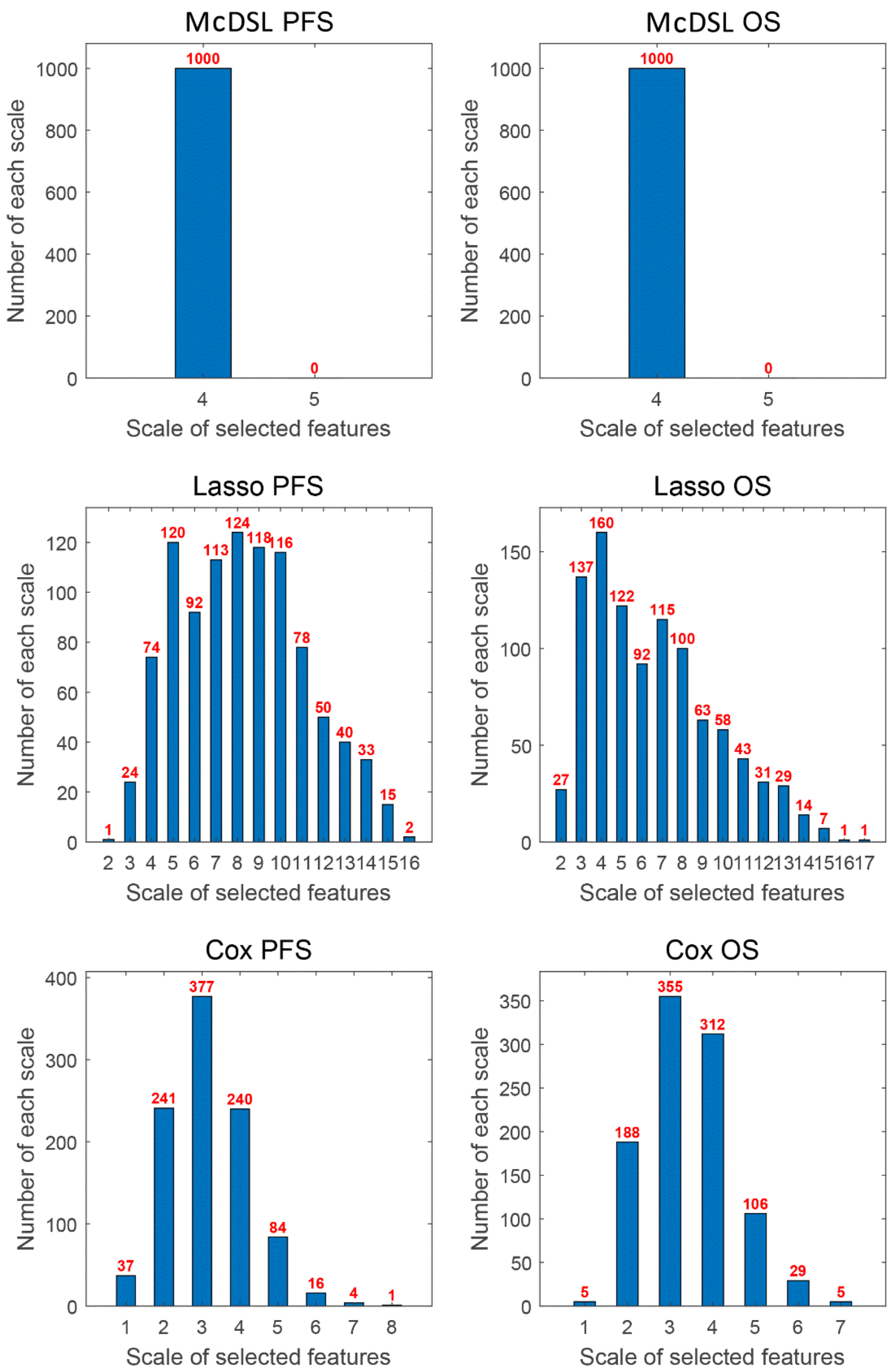

Figure S2: The importance ranking of variables in models based on McDSL, Lasso, and Cox model

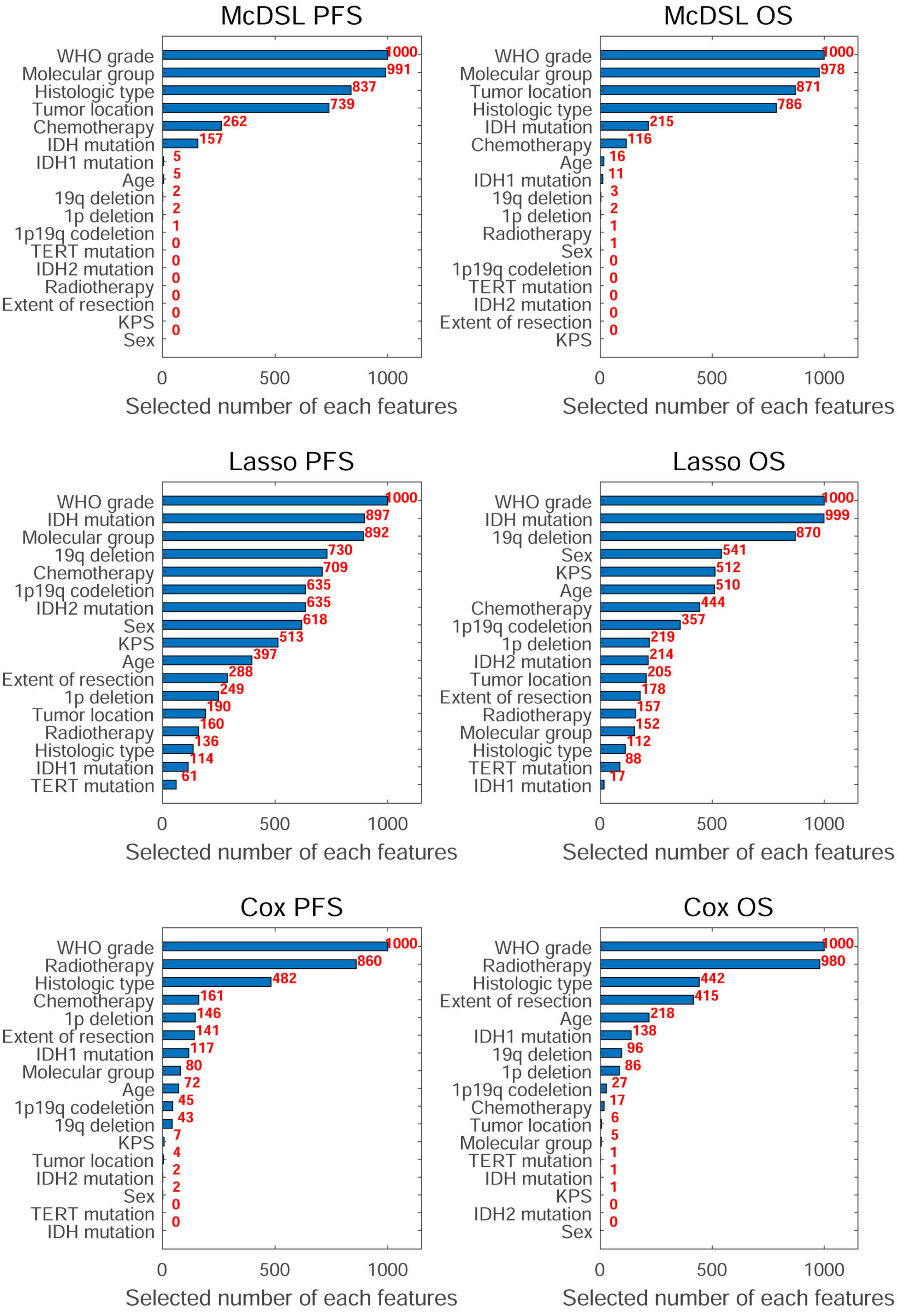

Supplement: Supplementary file 1 — Supplementary materials including figures. [file jcav12p1604s1.pdf]
